# An investigation of the factors that influence functional improvement in stroke rehabilitation

**DOI:** 10.3906/sag-2101-94

**Published:** 2021-06-28

**Authors:** Onur ALTUNTAŞ, Serkan TAŞ, Alp ÇETİN

**Affiliations:** 1 Department of Occupational Therapy, Faculty of Health Sciences, Hacettepe University, Ankara Turkey; 2 Department of Physiotherapy and Rehabilitation, Faculty of Health Sciences, Alanya Alaaddin Keykubat University, Antalya Turkey; 3 Department of Physical Medicine and Rehabilitation, Faculty of Medicine, Hacettepe University, Ankara Turkey

**Keywords:** Recovery, cerebrovascular accident, outcomes

## Abstract

**Background/aim:**

The purpose of this study was to determine effect of age, sex, affected extremity, disability severity, treatment type, cerebrovascular accident (CVA) etiology, number of treatment sessions, and CVA duration on the functional improvement of the stroke patients who participated in a physical medicine and rehabilitation program.

**Materials and methods:**

The research sample consisted of 322 stroke patients. Clinical and demographic features including age, sex, affected extremity, disability severity, treatment type, CVA etiology, number of treatment sessions, and CVA duration were recorded. Functional status was evaluated retrospectively by using the functional independence measure (FIM) at admission and discharge.

**Results:**

It was detected that discharge FIM score of the patients exhibited an increase of significance level (p < 0.05). It was found that age, number of treatment sessions, CVA duration and FIM admission score were determinative parameters in FIM gain level (p < 0.05) while sex, affected extremity, and CVA etiology were not effective in FIM gain level (p > 0.05).

**Conclusion:**

Results show that functional improvement after rehabilitation was better in the younger ages, shorter CVA durations and moderate functional disturbances. The findings obtained may be useful for stroke rehabilitation triage.

## 1. Introduction 

Stroke is defined by the World Health Organization as rapidly developing clinical signs of a focal disturbance that persist for at least 24 h or that lead to death with no apparent cause other than vascular origin [1]. It is a general health problem and ranks second among the major causes of death in adults. The mortality rate has decreased with advances in medical technology in the last decade and resulted in a dramatic increase in the number of stroke patients who require functional training [2]. According to World Health Organization data, stroke occurs in 15 million people around the world annually, of whom 5 million develop permanent disabilities. Stroke rehabilitation has therefore become the most important process in caring for stroke patients [2,3]. Rehabilitation processes play a major role in the optimal functional improvement of stroke patients [3–5].

Stroke patients show improvement after rehabilitation programs, however, the quality and rate of this improvement vary in stroke patients. Determination of factors such as age, sex, affected extremity, disability severity, and cerebrovascular accident (CVA) etiology and duration that affect functional improvement after stroke, and the prediction of improvement progression in line with these factors seem to have been popular research subjects for a long time [1,6–10]. However, it is seen that there are contradiction results in predictors of stroke outcome in literature. For example, some of these studies reported that age [11], sex [12], and CVA etiology [8] have an effect on the functional improvement of the stroke patients; however, the others indicated that age [13,14], sex [15], and CVA etiology [10] do not have an effect the functional improvement of the stroke patients. The studies are inadequate and have not enabled reaching a consensus. Prediction of rehabilitation result is crucial for the establishment of proper rehabilitation programs, correct patient assessment, informing the patients and their families about the potential for recovery according to the quality of care provided at home, and investigating new therapeutic strategies [6–10].

The purpose of this study was to determine the effects of age, sex, affected extremity, disability severity, treatment type, CVA etiology, number of treatment sessions, and CVA duration in stroke patients who participated in a physical medicine and rehabilitation program for functional improvement.

## 2. Materials and methods 

### 2.1. Patients and study design

This retrospective study included three hundred and twenty-two stroke patients attended the physical medicine and rehabilitation program both inpatients (n = 261) and outpatients (n = 61). Patients who experienced a new intracerebral hemorrhagic or ischemic stroke as inpatients or outpatients, those who had a history of CVA before the current one, those who had other neurological disorders, and those with no brain imaging data were excluded from the study. This study was approved by the ethics committee of Hacettepe University (GO 15/260), and was conducted in accordance with the rules of the Declaration of Helsinki. 

### 2.2. Procedure 

The patients were divided into 2 groups according to sex (male or female), affected extremity (right or left), CVA etiology (hemorrhagic or ischemic), and treatment type (inpatient or outpatient) in order to determine the effect of these parameters on stroke rehabilitation results. CVA etiology was determined by using imaging methods (brain computed tomography or magnetic resonance imaging). The patients were also divided into 4 groups according to CVA duration (1–3 months, 4–12 months, 1–2 years, and >2 years) in order to determine the effect of CVA duration on stroke rehabilitation results. The functional status of the patients was evaluated by using the functional independence measure (FIM) at the time of admission and discharge by clinicians trained in the use of the instrument. The FIM scale includes 18 items and measures independence in tasks involving self-care, sphincter control, mobility, locomotion, communication, and social cognition. Scores were assigned according to a 7-point Likert scale, and the score indicated the amount of assistance required to perform each item (7 = totally independent and 1 = totally dependent or not testable) [16,17]. The Turkish adaptation of the FIM used in this study has been shown to be reliable and valid in stroke patients [17]. Disability severity was stratified into 4 groups according to the total FIM admission score (<40, 41–60, 61–80, and >80) as described by Alexander et al. in order to determine the effect of the FIM admission score on stroke rehabilitation results [18].

### 2.3. Statistical analysis

Statistical analyses were performed by using the SPSS v: 15.0 software for Windows. Variables were investigated by using visual (histograms and probability plots) and analytical methods (Kolmogorov–Smirnov or Shapiro–Wilk test) to determine their distribution. As the demographic data and assessed parameters were nonnormally distributed, these parameters were presented using median and interquartile range. The Wilcoxon test was used to compare changes between FIM admission and discharge score. The Mann–Whitney U test was used to compare differences in clinical features according to sex, CVA etiology, affected extremity, and treatment type. The Kruskal–Wallis test was used to compare differences in clinical features according to CVA duration and disability severity. The correlation coefficients of the relationships between the parameters and their statistical significance were calculated by using the Spearman–Correlation Test. Correlation analysis results were interpreted as follows: 0.81–1.00 (very good correlation), 0.61–0.80 (good correlation), 0.41–0.60 (moderate correlation), 0.21–0.40 (fair correlation), and 0.00–0.20 (poor correlation). A multiple stepwise regression analysis was performed with FIM gain and FIM efficiency as dependent variables, and CVA etiology, sex, affected extremity, FIM admission score, treatment type, CVA duration, and age as independent variables. An overall 5% type 1 error level was used to indicate statistical significance. 

Based on the Kruskal–Wallis test results, in case of a difference between groups, to find out which group caused this difference, the Mann–Whitney U test was used to compare all the pairs. Statistical results were evaluated using the Bonferroni correction. Total statistical significance was set as type 1 percentage error 0.8% (5/6%). 

## 3. Results

The results of the patients who were divided into groups according to sex, affected extremity, CVA etiology, treatment type, disability severity, and CVA duration were given in Table 1. No statistically significant differences were found for age, CVA duration, number of treatment sessions, FIM gain and FIM efficiency levels, and FIM admission and FIM discharge scores when the patients were divided into 2 groups according to sex, affected extremity (right or left), CVA etiology (hemorrhagic or ischemic), and treatment type (outpatient or inpatient) (p > 0.05). The FIM discharge scores of the patients who were divided into 2 groups according to sex, affected extremity, CVA etiology, and treatment type increased significantly with respect to FIM admission score (p < 0.001).

**Table 1:** Results of the patients who were divided into groups according to sex, CVA etiology, sex, affected side, disability severity, number of treatment sessions, and CVA durationa.

		Age (years)	Duration (month)	Treatment sessions (n)	FIM admission	FIM discharge	FIMgain	FIMefficiency
Sex	Male (188, 58%)	63 (53–74)	3 (1–12)	20 (15–25)	73 (50–101)	93 (60–113) *	5 (0–19)	0.3 (0–0.9)
	Female (134, 42%)	68 (55–76)	4 (1–10)	20 (15–20)	73 (47–97)	92 (55–111) *	7 (1–19)	0.4 (0–.0.9)
Affected extremity	Right (141, 44%)	66 (53–75)	3 (1–9)	20 (15–20)	71 (48–95)	91 (56–110) *	7 (1–20)	0.4 (0–1.4)
	Left (181, 56%)	64 (55–74)	3 (1–12)	20 (15–25)	77 (50–101)	94 (61–114) *	5 (0–18)	0.4 (0.1–1.0)
CVA etiology	Hemorrhagic (72, 22%)	65 (53–75)	3 (1–9)	20 (15–25)	72 (46–95)	86 (56–109) *	7 (1–19)	0.4 (0.1–1.0)
	Ischemic (251, 78%)	65 (54–74)	4 (1–12)	20 (15–20)	75 (51–101)	96 (61–113) *	5 (0–18)	0.3 (0–0.8)
Type of treatment	Outpatient (61, 19%)	63 (52–73)	4 (2–11)	20 (15–20)	73 (51–95)	92 (58–111) *	9 (2–20)	0.5 (0.1–1.0)
	Inpatient (261, 81%)	66 (54–75)	3 (1–12)	20 (15–25)	73 (49–100)	92 (58–113) *	5 (0–18)	0.3 (0–0.9)
Disability severity	˂40 (49, 15%)	70 (59–76)	2 (1–5)	15 (10–20)	22 (20–30)	30 (22–40) *	3 (0–19)	0.3 (0–0.8)
	41–60 (77, 24%)	65 (55–74)	2 (1–5)	20 (15–25)	51 (46–55)	58 (52–77) *	8 (0–23)	0.3 (0–1.3)
	61–80 (57, 18%)	70 (59–76)	4 (1–8)	20 (15–25)	73 (67–75)	84 (75-100) *	10 (6–29) †	0.5 (0.3–1.2) †
	˃80 (139, 43%)	61 (49–73)	6 (2–18) ††	20 (15–20)	102 (93–117)	114 (102–122) *	4 (0-11)	0.2 (0–0.7)
CVA duration	0–3 months (150, 47%)	65 (53–74)	1 (1–2)	20 (15–25)	66 (45–95) §§	94 (56–110) *	13 (2–25) §	0.6 (0.1-1.4) §
	4–12 months (88, 27%)	66 (52–74)	5 (4-7)	20 (15–20)	70 (50–98)	82 (55–114) *	4 (0–14)	0.2 (0–0.7)
	1–2 years (32, 10%)	63 (53–73)	14 (13-18)	20 (15–20)	93 (79–112)	102 (92–113) *	4 (0–9)	0.2 (0–0.5)
	>2 years (52, 16%)	66 (57–75)	36 (27–72)	20 (15–20)	88 (56–114)	92 (61–115) *	2 (0–5)	0.1 (0–0.3)

FIM: functional independence measure, CVA: cerebrovascular accident.*p < 0.001, compared with the FIM admission score.†p < 0.001, compared with the groups with FIM scores of <40 and >80.††p < 0.008, compared with the groups with FIM scores of <40 and 61–80.§p < 0.001, compared with the groups with CVA durations of 4–12 months, 1–2 years, and ≥2 years.§§p < 0.001, compared with the groups with CVA durations of 1–2 and ≥2 years.a

CVA severity was classified into 4 groups. The results of the patients grouped according to CVA severity showed that the mean age of those with an FIM admission score of >80 was lower than that of the patients with an FIM admission score of ˂40 (p = 0.001) or 61–80 (p = 0.006). The mean CVA duration of the group with an FIM admission score of >80 was longer than those of the groups with FIM admission scores of <40 (p = 0.001) and 61–80 (p = 0.006). The FIM gain level of the patient group with an FIM score of 61–80 was higher than those of the groups with FIM scores of <40 (p = 0.001) and ˃80 (p < 0.001). Similarly, the FIM efficiency level of the patient group with an FIM score of 61–80 was higher than those of the groups with FIM scores of <40 (p = 0.003) and ˃80 (p < 0.001) (Table 1).

Evaluation of patient results according to CVA duration in the 4 groups at 0–3 months, 4–12 months, 1–2 years, and ≥2 years revealed that the FIM pretreatment score of the group with a CVA duration of 0–3 months was significantly lower those of the groups with durations of 1–2 years (p < 0.001) and ≥2 years (p = 0.001). The FIM admission score of the group with a CVA duration of 4–12 months was lower than that of the group with a CVA duration of 1–2 years. No significant difference in FIM discharge score was found among the 4 groups (p = 0.117). FIM gain and FIM efficiency levels were higher in the group with a CVA duration of 0–3 months than in the groups with CVA durations of 4–12 months (p < 0.001), 1–2 years (p = 0.001), and ≥2 years (p < 0.001) (Table 1).

A stepwise regression analysis was performed to determine which parameters had the strongest effect on the outcome in the FIM gain and FIM efficiency levels. Age, sex, affected extremity, CVA duration, number of treatment sessions, CVA etiology, treatment type and FIM admission score explained 34.1% of the total variance of the FIM gain level. The number of treatment sessions (15.2%), FIM admission score (6.1%), age (6.9%) and CVA duration (5.9%) were found to be determinant parameters of the FIM gain level, whereas sex, affected extremity, treatment type, and CVA etiology were not. On the other hand, age, sex, affected extremity, CVA duration, number of treatment sessions, CVA etiology, treatment type, and FIM admission score explained 21.4% of the total variance of the FIM efficiency level. FIM admission score (7.2%), age (8.5%) and CVA duration (5.7%) were found to be determinant parameters of the FIM efficiency level, whereas sex, affected extremity, treatment type, and CVA etiology were not.

The correlation analysis revealed negative and fair correlations between age and FIM admission score (r = −0.269, p < 0.001) and FIM discharge score (r = −0.360, p < 0.001). Fair correlations were also found between CVA duration and FIM admission score (r = 0.218, p < 0.001), FIM gain level (r = 0.354, p < 0.001), and FIM efficiency level (r = 0.345, p < 0.001). Similarly, a fair correlation was found between the number of treatment sessions and FIM gain level (r = 0.326, p < 0.001). A very good correlation was found between the FIM admission and FIM discharge scores (r = 0.902, p < 0.001), while negative and poor correlations were observed between the FIM admission score and FIM gain level (r = −0.158, p = 0.004), FIM efficiency level (r = −0.146, p = 0.009), and CVA duration (Table 2).

**Table 2:** Correlations between age, disease duration, number of treatment sessions, and functional measure scores.

	CVA duration	Treatment sessions (n)	FIM admission	FIM discharge	FIM gain	FIM efficiency
Age	0.016	–0.088	–0.269*	-–0.360*	–0.112	–0.104
CVA duration		–0.038	0.218*	0.041	–0.354*	–0.345*
Treatment sessions (n)				0.112*	0.326*	0.160*
FIM admission				0.902*	–0.158*	–0.146*

FIM: functional independence measure, CVA: cerebrovascular accident.*p ˂

## 4. Discussion

The purpose of this study was to investigate the effects of age, sex, affected extremity, disability severity, treatment type, CVA etiology, number of treatment sessions, and CVA duration on the functional improvement of stroke patients who participated in a physical medicine and rehabilitation program. The study results show that age was a predictive parameter of FIM gain and FIM efficiency levels, and negatively correlated with FIM admission and discharge scores. Lin et al. similarly reported a negative relationship between age and FIM discharge score [19]. Eskiyurt et al. also reported a negative correlation between age and total FIM score [11]. However, other studies in the literature did not find age to be an important factor of functional recovery. Luk et al. reported no relationship between age and functional recovery [13]. Similarly Wade et al. reported that age did not predict post-stroke functional gain level, whereas functional status on admission was a predictive factor [14]. 

The male and female patients in the present study showed similar functional improvements with stroke rehabilitation. Balcı et al. similarly reported no significant sex-related differences in FIM gain and FIM efficiency levels in patients who showed significant improvements with treatment [12]. Luk et al. also reported that sex had no effect on post-stroke functional loss and the rehabilitation results [15].

In this study, the proportion of patients with ischemic stroke was higher (78%) than that of patients with hemorrhagic stroke (22%), but the FIM gain and FIM efficiency scores of the patients with hemorrhagic stroke and those with ischemic stroke were similar. Cakir et al. evaluated the factors that influenced the FIM gain level in stroke patients and similarly reported that the etiology was cerebral ischemia in 80.9% and intracerebral hemorrhage in 19.1% of the patients, with similar FIM gain and FIM efficiency levels between the groups [20]. Jorgensen et al. also reported that CVA etiology did not influence neurological and functional improvements [10]. Nakipoğlu et al. reported that CVA etiology did not affect the rehabilitation results in stroke patients [21]. However, some studies in the literature reported conflicting results. Kelly et al. reported that patients with hemorrhagic stroke had more functional loss but showed more functional improvement after the rehabilitation program than patients with ischemic stroke [8]. Paolucci et al. found that patients with intracerebral hemorrhage showed better functional improvement than those with thromboembolic vascular disease [22]. 

The FIM gain levels were similar between the FIM admission and discharge scores, and the FIM efficiency level, between the patients with right and left extremity involvements in the present study. Similarly, Wade et al. reported that the side involved did not affect the rehabilitation results [14]. Yavuzer et al. reported that in patients whose right sides were involved, the initial functional scores were lower than those in patients whose left sides were involved, but rehabilitation gains were similar between the two groups [23]. Granger et al. reported that patients with left-side involvement had higher initial functional scores than those with right-side involvement [24]. Desrosiers et al. reported that the use of the nondominant extremity by right-side-dominant patients with right-side stroke could negatively affect their functionality [25]. The reason for the similar results in the patients with right- and left-side involvement could be the scale used to assess functional status. FIM assesses gross motor function and may therefore have overlooked losses in subtle motor abilities that might be caused by dominant extremity involvement.

The highest level of functional improvement during stroke rehabilitation was during the first 3 months after CVA in this study, and this improvement continued at a gradually decreasing rate over time. Individuals with longer CVA duration and better functional level on admission had relatively lower functional gain with a threshold effect. Similar to the present results, Öz et al. reported that patients with an early rehabilitation start had lower FIM admission scores than patients with a late start, but also experienced greater functional improvement [26]. Paolucci et al. reported a relationship between early start of rehabilitation and better functional improvement [22]. The present results revealed the importance of early rehabilitation applications and indicate that long-term follow-up of stroke patients may be beneficial.

Functional improvement was observed in all the groups, but the improvement in the patient with moderate physical disturbance (FIM score, 41–80) was better than that in patients with low (<40) or high FIM scores (>80). Inouye et al. divided their patients into 3 groups according to FIM admission score and found that the patients who were moderately affected at admission showed higher FIM gain levels than those who were severely affected [6]. Alexander included acute stroke patients in a 3-month rehabilitation program and found that functional gain levels were lower in the patients with low FIM scores (˂40) and that there was a higher chance of these patients to be directed to care centers [18]. Similarly, Ween et al. reported that the best functional gain after rehabilitation was in acute stroke patients with a less pronounced detrimental effect (FIM score ˃60) and that physical gain level was lower in individuals who were more severely affected (FIM score ˂40), resulting in increased rates of admission to care centers in the latter group [9]. 

The results of this study revealed that increased length of hospital stay or number of treatment sessions increased the FIM gain and efficiency levels. Ring et al. found length of hospital stay to be the best predictor of functional gain [27]. Gokkaya et al. found a significant relationship between FIM gain and length of hospital stay [28]. The present results could be interpreted as reflecting the FIM gain due to the prolongation of the rehabilitation process. Nevertheless, better functional improvement with longer treatment period may also be associated with the discharge criteria used in the authors’ clinic, where rehabilitation periods are prolonged when functional improvement is observed, and the patients are discharged when their functional improvement plateaus. The interpretation should be that “the treatment period was prolonged as functional improvement continued” rather than “the longer treatment period, the more functional improvement.”

This study revealed that whether the rehabilitation was provided in the inpatient and outpatient setting did not affect the rehabilitation results. The number of previous studies on the subject in the literature is inadequate. Inpatient treatment is preferred when patient follow-up is mandatory, access to the treatment center is difficult, loss of mobility is significant, and it is requested by the patients themselves. The disease duration, age, number of sessions, and FIM admission score of the inpatients and outpatients were similar in this study. The decision was made only by considering difficulties with access to treatment and the patients’ preferences. The rehabilitation results were therefore not affected, as expected.

This study has several limitations. First, it aimed to explain the effects of the rehabilitation process on functional improvement in relation to age, sex, affected extremity, etiology, treatment type, CVA severity, and CVA duration. The regression analysis revealed that age, sex, number of treatment sessions, CVA duration, FIM admission score, affected extremity, and CVA etiology explained 34.1% of the total variance in the FIM gain level. Medical complications (hypertension, shoulder pain, urinary tract infection, psychosocial problems, and cognitive problems) occurring during the stroke rehabilitation may set back any functional improvement, and including these factors in the present study could reveal other factors that could influence the rehabilitation success. Second, this study used the FIM, which assesses functional level and improvement in gross motor function. Any effects of the relevant factors on functional improvement might have been better revealed if subtle motor functions were assessed.

In conclusion, the functional improvement with rehabilitation was better in the stroke patients who were younger age and had shorter CVA durations and moderate functional disturbance. Sex, treatment type, affected extremity, and CVA etiology did not affect the rehabilitation results in these patients.

## Informed consent

The procedures followed were in accordance with the ethical standards of relevant institutional and consistent with the Helsinki Declaration. This study was approved by the ethics committee of Hacettepe University (GO 15/260). 
